# Endothelial angiogenic activity and adipose angiogenesis is controlled by extracellular matrix protein TGFBI

**DOI:** 10.1038/s41598-021-88959-1

**Published:** 2021-05-06

**Authors:** Seul Gi Lee, Jin Soo Kim, Ha-Jeong Kim, David D. Schlaepfer, In-San Kim, Ju-Ock Nam

**Affiliations:** 1 Department of Food Science and Biotechnology, Kyungpook National University, Daegu, 41566 Republic of Korea; 2 National Institute for Korean Medicine Development, Kyeongsan, 38540 Republic of Korea; 3 Department of Physiology, School of Medicine, Kyungpook National University, 680 Gukchaebosang-ro, Jung-gu, Daegu, 41944 Republic of Korea; 4 Moores Cancer Center, University of California, San Diego, La Jolla, CA 92093 USA; 5 KU-KIST Graduate School of Converging Science and Technology, Korea University, Seoul, 02841 Republic of Korea; 6 Biomedical Research Institute, Korea Institute of Science and Technology (KIST), Seoul, 02792 Republic of Korea

**Keywords:** Cell biology, Molecular biology

## Abstract

Several studies have suggested that extracellular matrix (ECM) remodeling and the microenvironment are tightly associated with adipogenesis and adipose angiogenesis. In the present study, we demonstrated that transforming growth factor-beta induced (TGFBI) suppresses angiogenesis stimulated by adipocyte-conditioned medium (Ad-CM), both in vitro and in vivo. TGFBI knockout (KO) mice exhibited increased numbers of blood vessels in adipose tissue, and blood vessels from these mice showed enhanced infiltration into Matrigel containing Ad-CM. The treatment of Ad-CM-stimulated SVEC-10 endothelial cells with TGFBI protein reduced migration and tube-forming activity. TGFBI protein suppressed the activation of the Src and extracellular signaling-related kinase signaling pathways of these SVEC-10 endothelial cells. Our findings indicated that TGFBI inhibited adipose angiogenesis by suppressing the activation of Src and ERK signaling pathways, possibly because of the stimulation of the angiogenic activity of endothelial cells.

## Introduction

Angiogenesis, the process of new blood vessel formation, plays an important role in obesity and adipogenesis by modulating multiple mechanisms^1^. This process is essential for facilitating the delivery of oxygen and nutrients into adipose tissues^2^. Consequently, a cascade of failed angiogenesis leads to adipose hypoxia and fibrosis, causing a rapid increase of adipose tissue and obesity^3^.

Adipose ECM molecules are associated with metabolically healthy adipose tissue and regulate inflammation, apoptosis, angiogenesis^4^, fibrosis, and subsequent metabolic deterioration^5,6^. Fibrosis is an excessive accumulation of ECMs, and can result from an imbalance between the synthesis and degradation of ECMs, such as collagens and fibronectin^4,7^. The maintenance of a high degree of flexibility of the ECM allows adipose tissue to expand in a healthy manner, without adverse metabolic consequences^4^. Previous studies have suggested that remodeling of the ECM through the deletion of the collagen VI gene results in the expansion of adipose tissue without the development of inflammation, accompanied by suppression of the activation of the MAPK signaling pathway^8,9^. From this perspective, targeting angiogenesis through the alternation of the ECM expression is a potential therapeutic strategy for metabolic diseases, such as obesity, diabetes, and angiogenesis-associated diseases. However, investigations on the role of ECMs in adipose angiogenesis and the resulting physiological changes remain limited to only a few ECMs.

Transforming growth factor-beta-induced protein (TGFBI, also known as βig-H3) is a secretory ECM protein induced by transforming growth factor-beta (TGF-β)^10,11^. TGFBI has been found to be expressed in several different cell types in a wide range of tissues^12,13^. TGFBI interacts with integrins and other ECMs to mediate cell adhesion and migration. A role for TGFBI has been reported in a wide range of physiological and pathological conditions, such as diabetic retinopathy, corneal dystrophy, and tumorigenesis ^14^. Some studies have revealed that TGFBI plays a role in various types of cancer, leading to tumor progression or inhibition by regulating angiogenic activity^15–17^. We have shown that TGFBI produces antiangiogenic activity in endothelial cells through direct interactions with αvβ3 integrin, leading to tumor inhibition^18^. In addition to its function as tumor microenvironment regulating factor, genetic polymorphisms in TGFBI are positively associated with levels of insulin and body mass index in the Korean population^19,20^. Additionally, TGFBI is present in human adipocytes, and the signaling pathway in which it participates could be involved in the differentiation of adipocytes^21^.

Altogether, these previous studies provide indirect evidence that TGFBI could contribute to adipose tissue expansion, which might be controlled by the angiogenic capacity. To date, however, the physiological role of TGFBI in an adipose angiogenesis has not been elucidated. Considering the role of TGFBI in tumor angiogenesis, adipocyte differentiation, and endothelial cells, we hypothesized that it may act as a regulator of angiogenic sprouting in adipose tissue.

In the present study, we showed that TGFBI controlled adipose angiogenesis and the angiogenic capacity of endothelial cells. We found that the deletion of TGFBI promoted the activation of Src and ERK in adipose tissue cells, while treatment of TGFBI protein inhibited the activation of these proteins in endothelial cells. These findings suggested that altered activation of Src and ERK could account for the modulation of endothelial cell functions by the absence or presence of TGFBI.

## Results

### The influence of TGFBI on the distribution of blood vessels in adipose tissue

**Figure 1:**
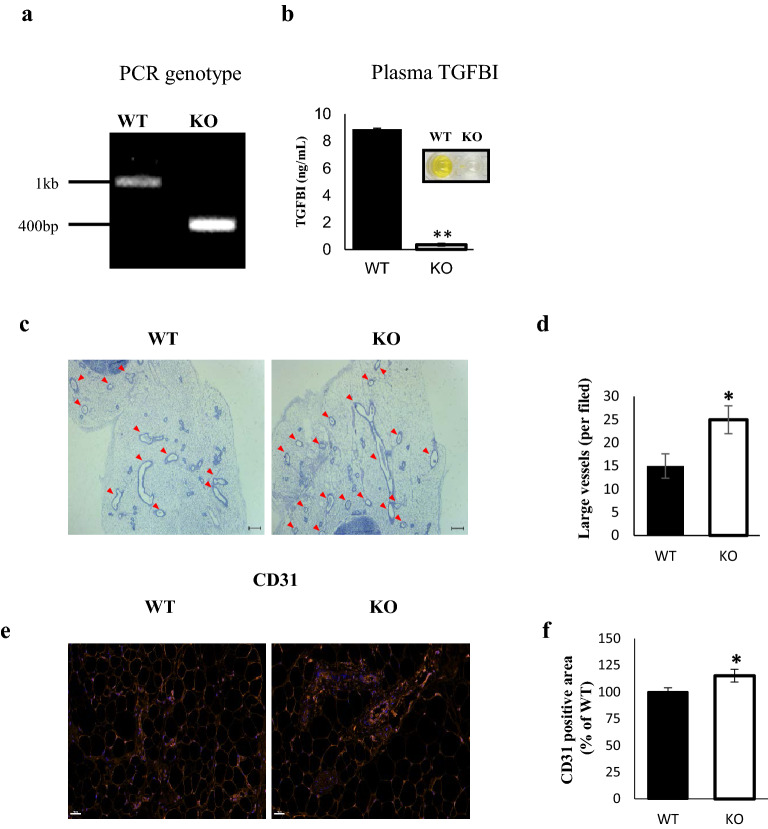
TGFBI KO mice show higher vessel density in adipose tissue compared with WT mice. **(a)** Genotyping was performed using PCR. The amplification targeted exon 3 of the TGFBI gene. **(b)** Plasma TGFBI concentrations in 20-week-old WT and KO mice (n = 3/group). **(c)** Representative H&E staining of iWAT sections from 20-week-old male WT and KO mice (n = 5/group). The red arrow indicates a large vessel (≥ 10 µm in diameter). Scale bars represent 200 μm. **(d)** The vessels were counted in sections from three mice, and expressed as mean number. **(e,f)** Representative images of CD31 immunohistofluorescence staining of iWAT from 20-week-old male WT and KO mice (n = 5/group). Scale bars represent 50 μm. **(f)** Quantification of area percentage of CD31 staining using ImageJ software. Data are presented as the mean ± S.E.M. *p < 0.05 versus WT.

To determine the role of TGFBI in adipose vascularity, we used wild type (WT) and TGFBI knockout (KO) C57BL/6 mice. The genotypes were confirmed by PCR using tail biopsy samples. WT mice gene showed a band at 1099 bp for TGFBI, while TGFBI KO mice showed a band at 376 bp (Fig. 1a). Using enzyme linked immunosorbent assay (ELISA), the expression of TGFBI in plasma was undetectable in the TGFBI KO mice (Fig. 1b). The mRNA expression of TGFBI was detected in inguinal white adipose tissue (iWAT) from WT mice (Supplementary Fig. 1a), although the relative expression was lower than that of liver tissue, which has been reported to express TGFBI abundantly ^22,23^.

Compared to WT mice, TGFBI KO mice exhibited a significantly increased large vessel density in iWAT (Fig. 1c,d). Although the capillary density was slightly reduced, the sum of the numbers of large vessels and capillaries in TGFBI KO mice was higher than those in WT mice (Supplementary Fig. 1b,c). This result suggested that TGFBI contributes to the regulation of adipose angiogenesis. Because angiogenesis is controlled by endothelial cells, we investigated whether TGFBI affects the deposition and population of endothelial cells in iWAT. Immunohistochemistry was performed using an anti-CD31 antibody that recognized a fixation-resistant epitope in endothelial cells expressed in both WT and KO mice. The expression of CD31 was stronger in iWAT from KO mice than it was in iWAT from WT mice. (Fig. 1e,f). Furthermore, we investigated the effect of TGFBI in other metabolic organ, such as the liver. Histological analysis demonstrated that the blood vessel density showed no differences between the livers of WT and KO mice (Supplementary Fig. 2a,b). Together, these results indicate that TGFBI deletion of TGFBI promoted adipose angiogenesis by regulating the population of endothelial cells, as assessed by CD31 staining.

**Figure 2:**
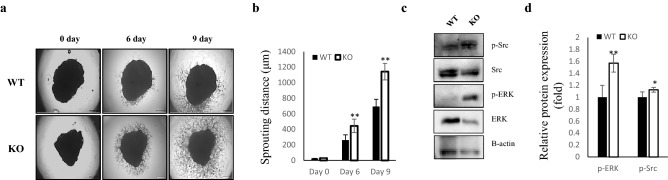
Enhanced capillary sprouting from adipose tissue in TGFBI KO mice. iWAT was dissected from 20-week-old male WT and KO mice and embedded on Matrigel. **(a)** Capillary sprouts emerging from iWAT were monitored for up to 9 days. Representative images of sprouted capillaries are shown at the indicated days. **(b)** The distance of capillary sprouting was quantified with Image J software**.** **(c)** Cells were isolated from capillary branches on day 9 post-embedding. Total proteins were extracted as described in the “Materials and methods” section. Representative expression of the indicated proteins in isolated primary cells from capillary sprouts in WT and KO mice (n = 3/group). **(d)** Relative expression of these proteins. Data are presented as the mean ± s.e.m. *p < 0.05, **p < 0.01 versus WT.

### Deletion of TGFBI enhances capillary sprouting from adipose tissue and activates MAPK-activated protein kinases

Based on results showing increased vessel density in TGFBI KO mice, we speculated that deletion of TGFBI could facilitate the activation of vessel sprouting in an ex vivo tissue culture system. After 6 days of embedding adipose tissue in Matrigel, sprouted capillaries in iWAT explants from KO mice were comparable to that of WT mice. Although capillaries emerged from iWAT in both WT and KO mice at days 6 and 9 post-embedding, the distance was remarkably increased in KO mice compared with that of WT mice during the same time period (Fig. 2a,b). The distance of capillaries from iWAT was also significantly increased in KO mice in a time-dependent manner, which made it somewhat difficult to identify all sprout formation.

Next, we investigated the mechanism through which TGFBI deletion promotes capillary sprouting from iWAT. We isolated cells from sprouted capillaries and analyzed the activity of the MAPK signaling pathway. The expression of phosphorylated Src and Erk was up-regulated in the isolated cells from TGFBI KO mice, approximately 1.5-fold and 1.2-fold, respectively, compared with that of WT mice (Fig. 2c,d). These observations indicated the TGFBI deletion enhances the ability of capillary sprouting in adipose tissue, which may be regulated by the activation of MAPK signaling pathway.

### TGFBI inhibits tube formation and migration activity stimulated by adipocyte-conditioned medium in SVEC-10 endothelial cells

**Figure 3:**
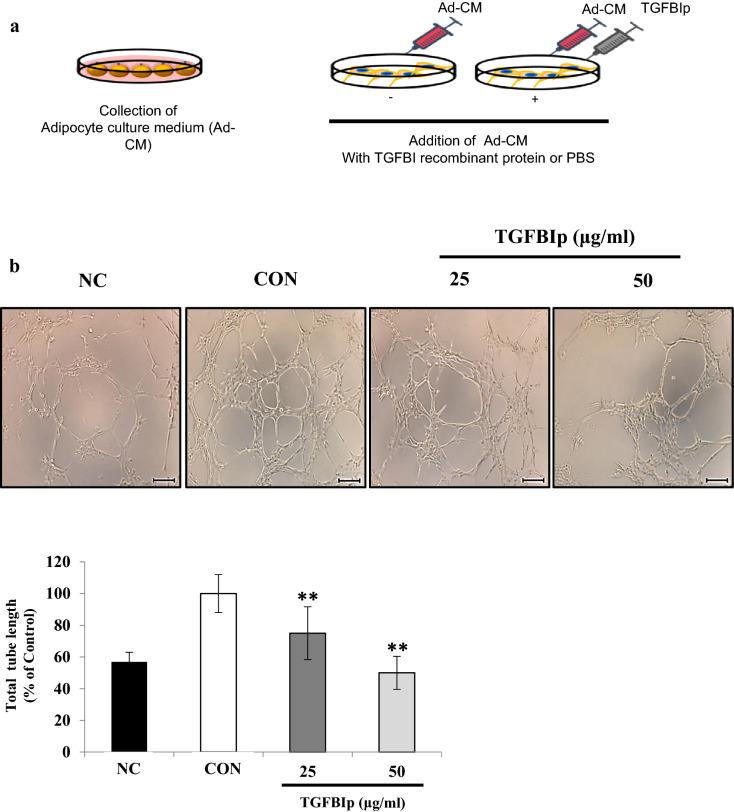

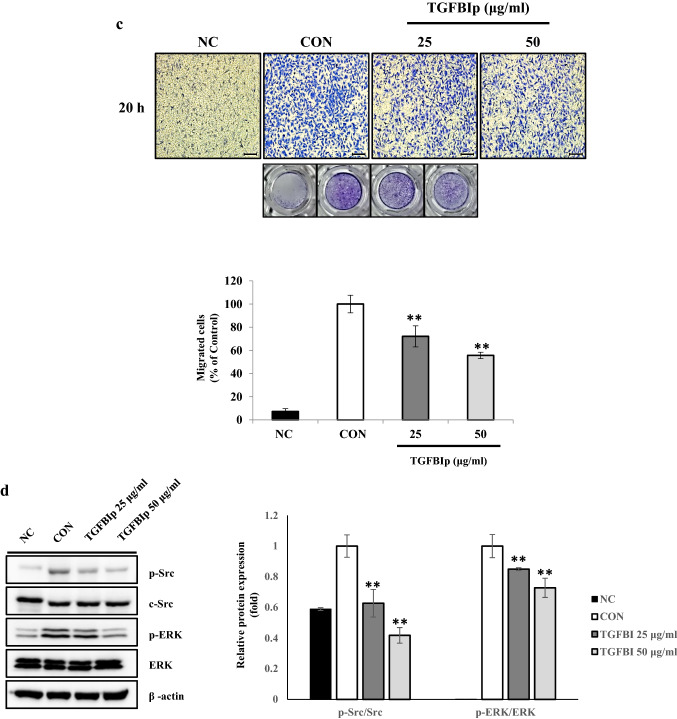
TGFBI reduces the migration and tube formation activity stimulated by Ad-CM in SVEC-10 endothelial cells. **(a)** Schematic of the in vitro experiment using SVEC-10 cells treated with Ad-CM. Negative control cells (NC) were cultured in normal growth medium with the same amount of PBS. **(b)** Representative images of tube formation (upper) and quantitation of the total tube length (lower). Scale bars represent 200 μm. **(c)** Representative images of migration (upper) and quantitation of the migrated cells after staining with crystal violet (lower). (d) SVEC-10 cells were starved overnight, treated with TGFBI for 60 min, and then stimulated with adipocyte-CM for 10 min. Expression levels of the indicated proteins were quantified (right). Data are presented as the mean ± s.e.m. *p < 0.05, **p < 0.01 versus control.

Since our data revealed that TGFBI induced overexpression of endothelial cell markers in adipose tissue (Fig. 1e,f), we hypothesized that TGFBI may affect the angiogenic activity of endothelial cells in response to adipokines. Therefore, we incubated SVEC-10 mouse endothelial cells in Ad-CM with recombinant TGFBI protein (TGFBIp) to determine the effects on tube formation and migration (Fig. 3a). We confirmed that Ad-CM promoted tube formation and migration of SVEC-10 in endothelial cells compared with control cells incubated under normal culture conditions; this group is referred to as negative control, NC (Fig. 3b,c). Treatment with TGFBIp significantly inhibited tube formation and migration activity of SVEC-10 cells compared with control cells treated with Ad-CM alone (CON). Tube formation activity was reduced by 50.0% and 23.6% when cells were treated with TGFBIp at concentrations of 25 and 50 μg/ml, respectively (Fig. 3b). In addition, no difference was observed in the tube formation activity between NC and NC with TGFBI treatment groups (Supplementary Fig. 3). This observation suggested that TGFBI does not affect the angiogenic capacity and has no apparent cytotoxicity on SVEC-10 endothelial cells cultured under normal conditions.

In the migration assay, reduced activity resulting from the same treatment of TGFBIp was 72.1% and 55.7%, respectively (Fig. 3c). Furthermore, the expression level of phosphorylated ERK and Src was significantly lower in TGFBIp-treated SVEC-10 cells and was dose-dependent (Fig. 3d). The 0.4- and 0.8-fold decrease in p-Src and p-ERK, respectively, following treatment with 50 μg/ml of TGFBI. Collectively, these data suggest that TGFBI suppressed the activation of Src and ERK in SVEC-10 endothelial cells stimulated with Ad-CM, and this suppression could contribute to regulating the angiogenic functions of SVEC-10 endothelial cells, such as tube formation and migration.

### Deficiency of TGFBI modulates vessel infiltration into Matrigel containing Ad-CM

**Figure 4:**
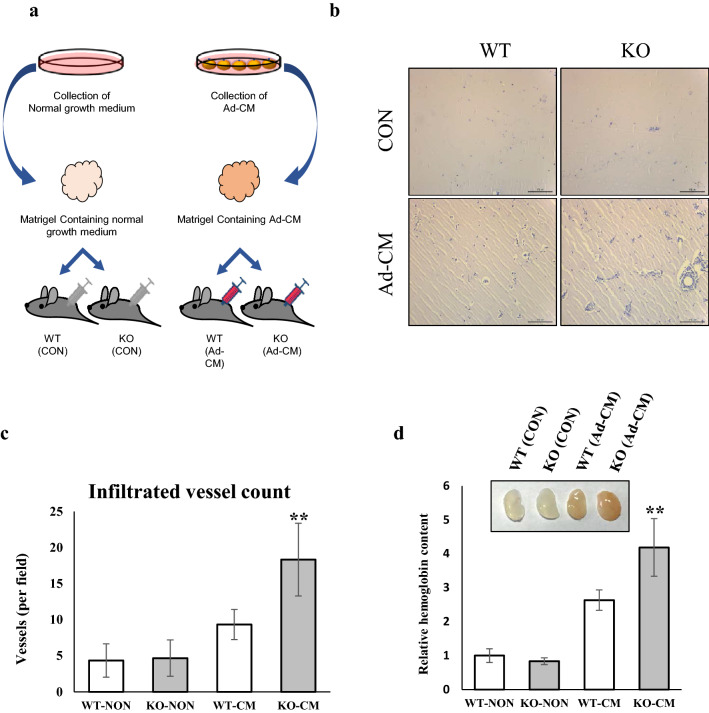
TGFBI modulates vessel infiltration into Matrigel containing Ad-CM. Matrigel impregnated with either normal medium or Ad-CM was injected in WT and KO mice. Normal medium: DMEM-high glucose, 10% FBS, and 1% antibiotics. **(a)** Schematic of the Matrigel plug assay in WT and TGFBI KO mice treated with Ad-CM. **(b)** Representative H&E staining section of Matrigel from WT and KO mice (n = 3/group). Scale bars represent 100 μm. **(c)** The infiltrated vessels were counted in three Matrigel H&E-stained sections and expressed as the mean number. Significant differences were evaluated between two groups of experiments in the same condition (WT-NON versus KO-NON or WT-CM versus KO-CM). **(d)** Relative hemoglobin content of Matrigel plugs. The insert image represents Matrigel for each group. Data are presented as the mean ± s.e.m. **p < 0.01 versus control.

To ensure that TGFBI is responsible for adipose angiogenesis in animals, we examined the effect of TGFBI on vessel infiltration into Matrigel with Ad-CM. Matrigel was mixed with either normal medium (CON) or Ad-CM, and the mixture was injected subcutaneously in mice (Fig. 4a). The addition of Ad-CM promoted vessel infiltration from the surrounding adipose tissue into the Matrigel (Fig. 4b–d). We observed that Matrigel containing Ad-CM injected into TGFBI KO mice exhibited a higher vessel density and hemoglobin content compared with that of WT mice. In contrast, there was no significant difference between WT and KO mice injected with Matrigel containing normal medium. These results indicated that Ad-CM promotes angiogenesis and the deletion of TGFBI has a positive impact on angiogenesis.

## Discussion

Adipose expansion can be limited by its vascular supply, raising the possibility that the angiogenic potential of specific adipose tissues may be critical to limiting their maximal expandability^24^. Several lines of evidence indicate that overexpression of VEGF in adipose tissues results in increased vascularization and decreased inflammation, which eventually leads to protection from HFD-induced obesity and insulin resistance^24,25^. Moreover, it has been previously noted that the expression of angiogenic factor is closely related to environmental conditions such as ECM components and abundance^26^. For example, the ECM protein, periostin (Postn), contains four tandem repeats of the FAS1 domain, which are also present in TGFBI^27,28^. A previous study shown that Postn contributes to HFD-induced adipose tissue inflammation and fibrosis^29^. However, the mechanism through which ECM influences the pathology of adipose tissue is poorly understood.

In this study, we discovered that TGFBI regulates adipose angiogenesis through a Src-MAPK signaling-dependent mechanism. Angiogenesis is accelerated with activating multiple signal pathways, such as the MEK/ERK pathway for endothelial cell proliferation and the p125^FAK^/Src/p38 MAPK signaling system for endothelial cell migration ^30^. Src, a membrane-associated nonreceptor tyrosine kinase, have a crucial role in signal transduction downstream of several growth factor receptors including VEGFR^31^. Its downstream transduction pathways include ERK signaling, is the most classical pathway of MAPK pathways ^32^. The activation of these proteins therefore could be responsible for the proliferation and angiogenic activity of endothelial cells induced by pro-angiogenic factors ^33^.

In fact, TGFBI-deficient mice showed increased vessel density and CD31 positive area in adipose tissue. Moreover, we found that TGFBI-deficient mice dramatically promoted vessel sprouting in iWAT and activation of Src and ERK proteins was observed. Conversely, when the endothelial cells were treated with TGFBIp, the activation level of these proteins was reduced and the angiogenic activity was diminished in these cells. These data suggest that TGFBI contributes to the progression of adipose angiogenesis by regulating endothelial cell function in response to adipokines, which are present in Ad-CM.

Angiogenesis is associated with chronic conditions in most organs and the development and progression of cancerous diseases^34^. An excessive accumulation of the ECM and an altered angiogenic balance in the liver result in the disruption of the normal liver structure and function^35^. Here, we showed that TGFBI mRNA abundance was higher in the liver than in adipose tissue. There was no obvious difference in vessel density between the livers of WT and TGFBI KO mice. This result implied that the TGFBI-specific contribution to angiogenesis might be context dependent, although it was expressed abundantly in the liver.

Adipose browning is a process in which white adipocytes can change their phenotype and function as brown-like adipocytes. This represents a promising therapeutic strategy for the treatment of various metabolic diseases such as diabetes and obesity^36,37^ According to previous studies, the WAT vasculature plays an essential role in controlling adipokine release and adipose angiogenesis. These vasculatures may ultimately facilitate adipose browning, resulting in metabolic health^38,39^. Given the contribution of TGFBI to adipose angiogenesis in the current study, we suggest that TGFBI may function as a regulator of chronic conditions, such as expansion, inflammation, and browning of WAT, although there is no direct evidence yet.

Overall, the results from our study suggest that TGFBI plays a significant role in adipose angiogenesis by regulating essential processes in endothelial cells. To our knowledge, these finding are the first to support a role for TGFBI in adipose angiogenesis, possibly through activation of the MAPK signaling pathway.

## Materials and methods

### Reagents

Dulbecco’s Modified Eagle Medium (DMEM), fetal bovine serum (FBS), and newborn calf serum (NBCS) were purchased from Gibco Life Technologies (Grand Island, NY, USA). Insulin, indomethacin, dexamethasone, and 3-isobutyl-1-methylxanthine (IBMX) were purchased from Sigma-Aldrich (St Louis, MO, USA). The plasma and cell supernatant TGFBI content were determined using the mouse beta IG H3 ELISA kit (Abcam, Cambridge, MA, USA). Transwell chamber inserts ((polycarbonate membrane, 8 μm pore size) and Matrigel were purchased from Costar (MA, USA) and Corning (NY, USA), respectively.

### Animals

The heterozygous TGFBI + / − mice were bred to generate TGFBI − / − (KO) and TGFBI + / + (WT) littermates as previous described^40^. The targeting vector containing PGK-neomycin flanked by FLP recombinase target sequences was microinjected into C57BL/6 blastocysts, and then the FLP recombinase target-flanked PGK-neomycin cassette was removed by Flp-mediated recombination. To delete exon 3 of the TGFBI gene, floxed-TGFBI mice were crossed with protamine I-Cre (PrmI-Cre) transgenic mice to generate TGFBI + / − mice. The gene targeting strategy is shown in Supplementary Fig. 4. The genotypes were determined from tail biopsies using polymerase chain reaction (PCR). The primers used were as follows: sense (5′-CCATACTCTGACTTCCAGGTTATTA-3′) and antisense (5′-TGGCAGACTAGCAAGGGTTT-3′). Mice were maintained in an environment with 10–20% humidity, 24 ± 1 ℃ temperature, and a 12-h light–dark cycle. The mice were fed a standard diet supplemented with 10% fat. All animal experiments were approved by the Institutional Animal Care Committee of the Kyungpook National University (approval number: KNU 2016-0070). The study was carried out in compliance with the ARRIVE guidelines. All methods were performed in accordance with the relevant guidelines and regulations.

### Recombinant TGFBI

Recombinant TGFBIp was generated by expressing TGFBIp in *Escherichia coli* as previously described ^17,41^. Briefly, TGFBI cDNA was cloned into pBluescript and inserted into the EcoRV and EcoRI sites of pET-29b (Novagen). A clone was selected and cultured with 1 mM IPTG to induce TGFBI protein expression. The inclusion bodies were dissolved in 8 M urea buffer containing 0.5 M NaCl, 5 mM Imidazole, and 20 mM Tris–HCl (pH 7.8). The his-tagged TGFBI protein was purified using Ni–NTA resin and dialyzed. Purified recombinant protein was subjected to SDS–PAGE and visualized with Coomassie brilliant blue staining solution.

### Cell culture and conditioned medium preparation of 3T3-L1 adipocytes

SVEC4-10 endothelial and 3T3-L1 preadipocytes were maintained in DMEM supplemented with either 10% FBS or 10% newborn calf s**e**rum (NBCS), respectively, and incubated at 37 °C in a humidified 5% CO_2_ atmosphere. For adipocyte differentiation, 3T3-L1 preadipocytes were exposed to a differentiation medium (MDI) consisting of DMEM containing 10% FBS, 0.5 mM IBMX, 1.72 nM insulin, 1 µM dexamethasone, and 100 µM indomethacin for 48 h. Afterwards, stimulated cells were maintained in medium supplemented with 10% FBS and 1.72 nM insulin for an additional 6 days by replacing half of the medium every 2 days. Eight days after induction of differentiation, the culture medium of the mature 3T3-L1 adipocytes was collected and centrifuged at 500 xg for 10 min at 4 °C to remove cellular debris. The resulting supernatant was used for experiments requiring adipocyte-conditioned medium (Ad-CM).

### Tube formation and transwell migration assay

Tube formation and Transwell migration assays were performed using SVEC4-10 murine endothelial cells as previously described^42^. Briefly, Matrigel was added to a 96-well plate and allowed to polymerize at 37 °C for 15 min. SVEC4-10 cells were either untreated or treated with various concentrations of TGFBI (25, 50 μg/ml) in Ad-CM for 30 min. Cells (5 × 10^4^) were seeded into Matrigel-coated plates and incubated at 37 °C for 6 h. The formation of tube structures was confirmed and imaged with a microscope. The total tube lengths were quantified using ImageJ software and expressed as percentages.

For the Transwell migration assay, SVEC4-10 cells were untreated or treated with various concentrations of TGFBI (25, 50 µg/ml) in Ad-CM for 30 min. Cells (2 × 10^4^) were seeded into the upper chamber of a Transwell unit and allowed to migrate for 20 h at 37 °C. After that, the inserts were cleaned with cotton swabs, fixed with 6.0% glutaraldehyde, and stained with 0.1% crystal violet. Migrated cells were imaged and counted using ImageJ software.

### Ex vivo angiogenesis assay of adipose tissue

Inguinal white adipose tissue (iWAT) was freshly harvested from WT and KO mice (8-week-old mice). Each iWAT was cut into 1 mm^3^ pieces, embedded in Matrigel in 96-well plates, and cultured in EBM-2 medium (Lonza, Walkersville, MD, USA) for 9 days at 37 °C. The medium was replaced every 2 days and angiogenic sprouting was observed by microscopy at the indicated times. The sprouting distance from adipose tissue was calculated using ImageJ software.

The isolation of cells comprising capillary branches was performed for further analysis by Western blotting as described previously^43^. At 9 days post-embedding, the EBM-2 culture medium was removed and washed with phosphate-buffered saline (PBS), and dispase (cat. no. 354235; BD Biosciences, San Jose, CA) was added to digest the Matrigel for 2 h at 37 °C. Then, detached and floating cells were confirmed under a microscope, and the digested Matrigel was removed by centrifugation at 200 × *g* for 10 min at room temperature. The supernatant was aspirated, and the cell pellet was resuspended in cell lysis buffer to prepare the samples for subsequent Western blot analysis.

### Real-time reverse transcription polymerase chain reaction (RT-PCR)

Total RNA was extracted from iWAT and liver in WT mice using the RNeasy Lipid Tissue Mini Kit (Qiagen**,** Hilden, Germany) according to the manufacturer's instructions. Reverse transcription and cDNA synthesis were performed using a first strand cDNA synthesis Kit (Toyobo, Osaka, Japan). RT-PCR was performed with an iCycler iQ Real-Time PCR Detection System (Bio-Rad Laboratories, USA) using SYBR Green (Toyobo, Japan). All primers were synthesized by Macrogen (Seoul, Korea) and the primer sequences are shown in Table 1.

**Table 1:** Primer sequences used for quantitative RT-PCR.

Gene	Forward primer	Reverse primer
TGFBI	GGAAGCTTCACCATCTTTGC	ATGTTGACGTTGCTCACCAG
β-actin	CGTGCGTGACATCAAAGAGAA	GCTCGTTGCCAATAGTGATGA

### Western blot analysis

Western blot analysis was performed as described previously^44^. Briefly, the tissues and cells were harvested and lysed in RIPA buffer. The protein content was measured using the Bradford assay. Equivalent amounts of protein were subjected to 7.5–10% SDS-PAGE and transferred onto nitrocellulose membranes. The membranes were blocked with 5% non-fat skim milk and incubated with the following primary antibodies p-Src, Src, p-ERK, ERK (Cell Signaling Technology, Beverly, USA), and β-actin (Santa Cruz Biotechnology, CA, USA). Subsuquently, the membranes were incubated with HRP-conjugated anti-mouse or anti-rabbit secondary antibodies (Santa Cruz Biotechnology) for 1 h at room temperature. Membranes were then washed with a solution of tris-buffered saline and Tween 20 (10 mmol/l Tris, pH 8.0, 150 mmol/l NaCl, and 0.05% Tween 20), and developed using enhanced chemiluminescence (GE Healthcare, Buckinghamshire, UK). All images of the original blots are shown in Supplementary Figs. 5 and 6.

### Matrigel plug assay

Matrigel (0.3 ml) containing 25% (v/v) Ad-CM and 10 units heparin was injected subcutaneously into WT and KO mice (10-week-old male). After 3 weeks, the Matrigel plugs were removed and stained with hematoxylin and eosin (H&E) to observe the formation of new microvessels. To quantify the blood vessel density, all sections were imaged under a microscope. The infiltrated vessels in the section were observed and counted.

In an ongoing experiment, the hemoglobin content of the removed Matrigel plug was measured using Drabkin’s method as previously described^45^. Briefly, the Matrigel plugs were homogenized in distilled water containing 0.2% heparin overnight at 4 °C. The lysate was centrifuged at 5000×*g* for 20 min and the supernatant was incubated with Drabkin’s reagent (Sigma, MO). The absorbance was measured using spectrophotometry (Tecan, Austria GmbH, USA) at 540 nm.

### Immunohistofluorescence (IHF)

iWAT was harvested from WT and KO mice, fixed with 4% PFA, and embedded in paraffin. The embedded tissues were cut into 5 μm-thick sections and stained with H&E. To quantify the blood vessel density, all sections were photographed under a microscope. The blood vessels were categorized into large vessels and capillaries according to their sizes. The large vessel and capillary densities were presented as the number of those per photographed image.

To determine the presence and expression of CD31 in adipose tissue, 4-μm sections of specimens were cut from formalin-fixed paraffin-embedded (FFPE) blocks. Slides were heated for at least one hour in a dry oven at 60℃, then followed by multiplex immunofluorescence staining with a Leica Bond Rx Automated Stainer (Leica Biosystems, Newcastle, UK). Briefly, the slides were baked for 30 min and dewaxed with Leica Bond Dewax solution (#AR9222, Leica Biosystems), followed by antigen retrieval with Bond Epitope Retrieval 2 (#AR9640, Leica Biosystems) in a pH 9.0 solution for 30 min. The primary antibodies for CD31 (ab124432, Abcam, dilution 1:500) were incubated for 30 min in a humidified chamber at room temperature, followed by detection using the Polymer HRP Ms + Rb (ARH1001EA, AKOYA Biosciences) for 10 min. Visualization of CD31 was accomplished using Opal 690 TSA (dilution 1:150) for 10 min, after which the slide was treated Bond Epitope Retrieval 1 (#AR9961, Leica Biosystems) for 20 min to remove bound antibodies before the next step in the sequence. Nuclei were subsequently visualized with DAPI, and the section was coverslipped using ProLong Gold antifade reagent (P36934, Invitrogen). To quantify the CD31 staining area, all sections from three mice per experimental group were selected. The immunostaining area of CD31 was measured using ImageJ software, as previously described^46^. The red channel was separated from blue, converted to greyscale, and the background was subtracted. The values of the monochromatic immunofluorescent stacks were then measured and expressed as means of area percentage.

### Statistical analysis

The data were expressed as means ± SEM and statistical analyses were performed using SPSS 23 software (SPSS, Chicago, IL, USA). The differences between the means of groups were analyzed by an unpaired Student’s *t*-test or by one-way analysis of variance. Values of p < 0.05 were considered statistically significant. All in vitro and in vivo animal experiments were repeated three times.

## Supplementary Information

Supplementary Figures.
