# Supplementary material for: Endothelial angiogenic activity and adipose angiogenesis is controlled by extracellular matrix protein TGFBI
Source: Sci Rep. 2021 May 6;11:9644. doi: 10.1038/s41598-021-88959-1 (PMC8102489; doi:10.1038/s41598-021-88959-1)
Supplement: Supplementary file 1 — Supplementary Figures. [file 41598_2021_88959_MOESM1_ESM.pptx]

## Slide 1
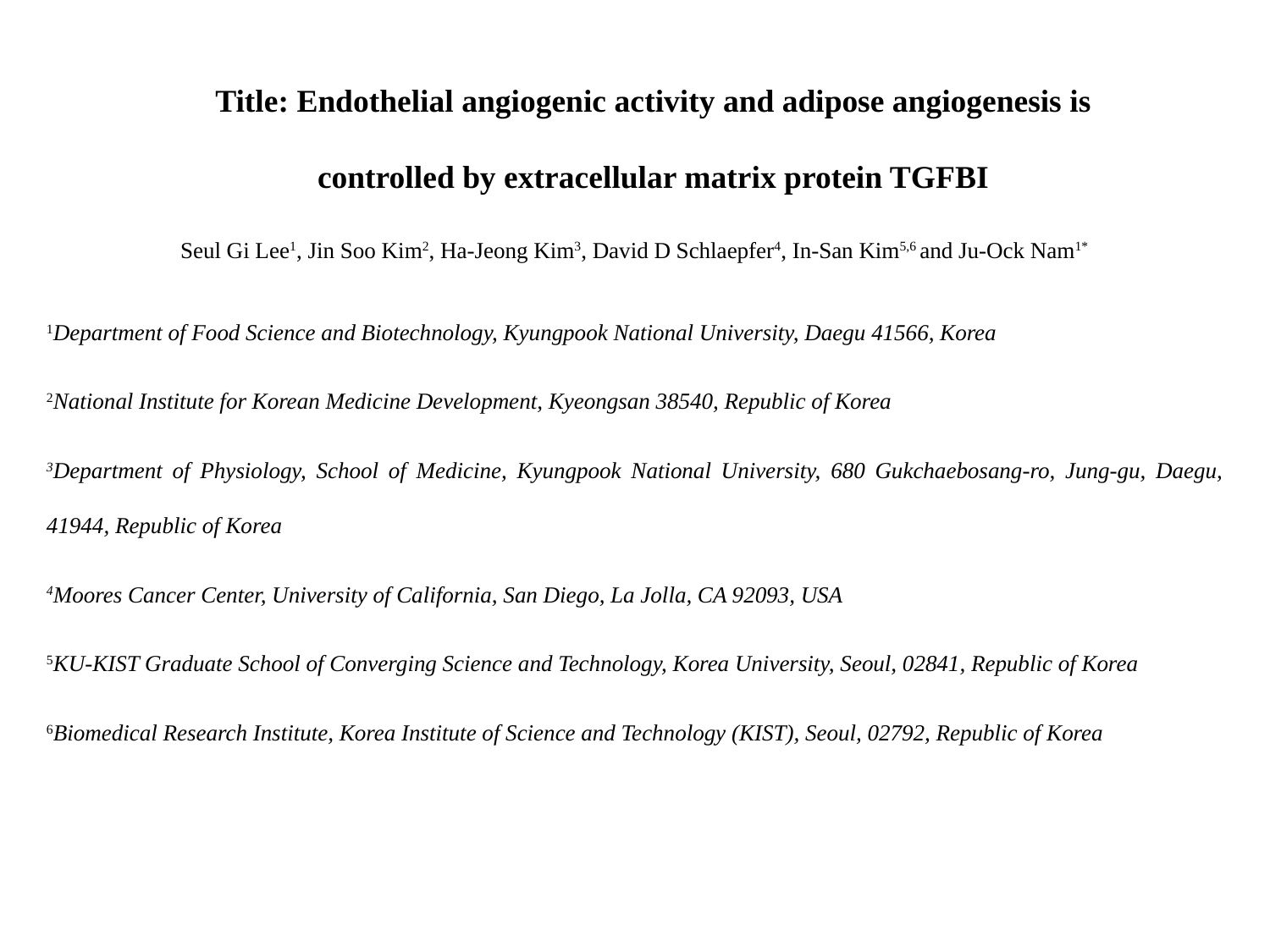

Title: Endothelial angiogenic activity and adipose angiogenesis is controlled by extracellular matrix protein TGFBI
Seul Gi Lee1, Jin Soo Kim2, Ha-Jeong Kim3, David D Schlaepfer4, In-San Kim5,6 and Ju-Ock Nam1*
1Department of Food Science and Biotechnology, Kyungpook National University, Daegu 41566, Korea
2National Institute for Korean Medicine Development, Kyeongsan 38540, Republic of Korea
3Department of Physiology, School of Medicine, Kyungpook National University, 680 Gukchaebosang-ro, Jung-gu, Daegu, 41944, Republic of Korea
4Moores Cancer Center, University of California, San Diego, La Jolla, CA 92093, USA
5KU-KIST Graduate School of Converging Science and Technology, Korea University, Seoul, 02841, Republic of Korea
6Biomedical Research Institute, Korea Institute of Science and Technology (KIST), Seoul, 02792, Republic of Korea

## Slide 2
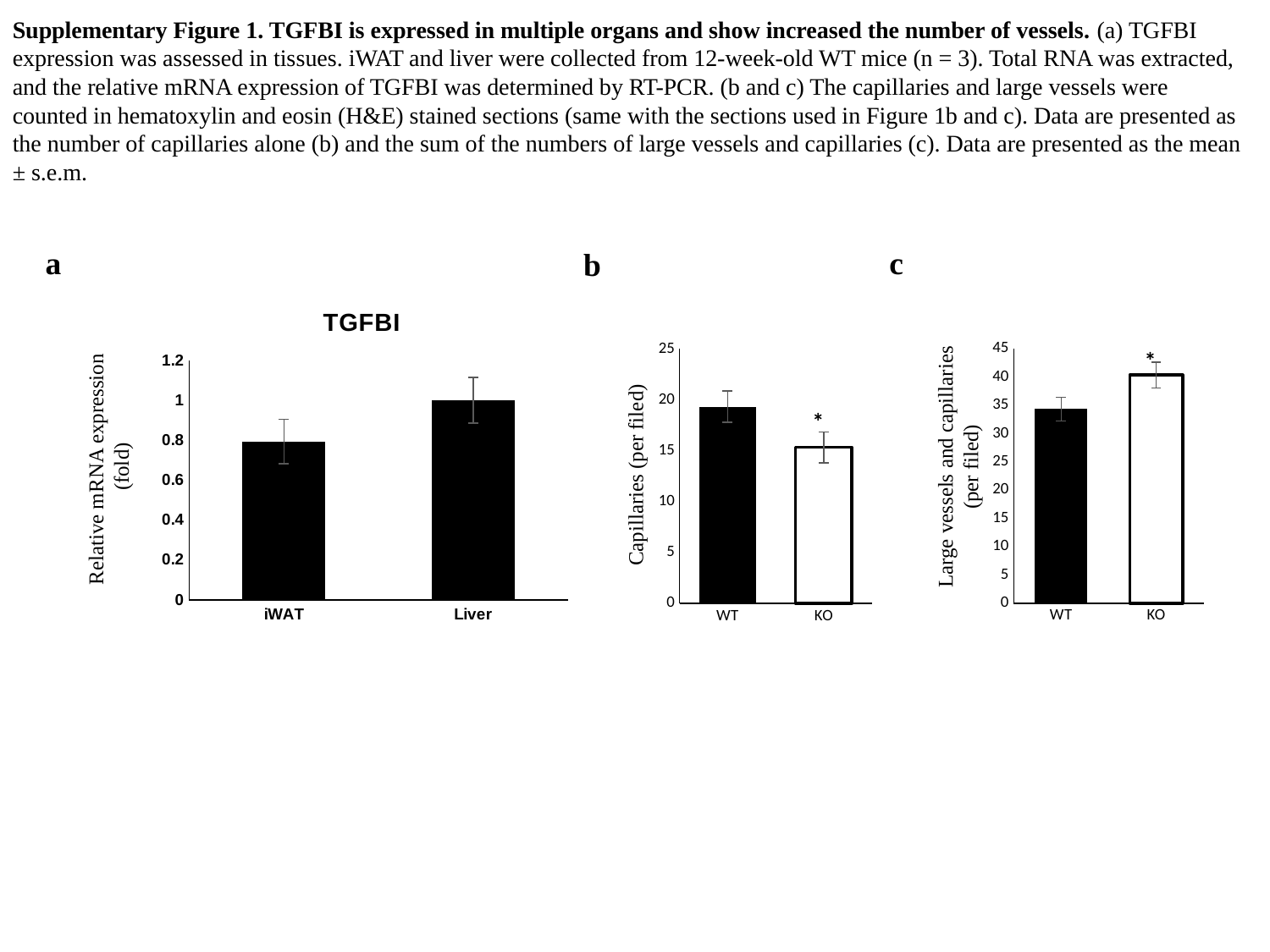

Supplementary Figure 1. TGFBI is expressed in multiple organs and show increased the number of vessels. (a) TGFBI expression was assessed in tissues. iWAT and liver were collected from 12-week-old WT mice (n = 3). Total RNA was extracted, and the relative mRNA expression of TGFBI was determined by RT-PCR. (b and c) The capillaries and large vessels were counted in hematoxylin and eosin (H&E) stained sections (same with the sections used in Figure 1b and c). Data are presented as the number of capillaries alone (b) and the sum of the numbers of large vessels and capillaries (c). Data are presented as the mean ± s.e.m.
a
c
b
### Chart: TGFBI
| Category | |
|---|---|
| iWAT | 0.79224 |
| Liver | 1.0 |Relative mRNA expression
(fold)
### Chart
| Category | |
|---|---|
| WT | 34.33333333333333 |
| KO | 40.333333333333336 |
### Chart
| Category | capillaries |
|---|---|
| WT | 19.333333333333332 |
| KO | 15.333333333333334 |*
*
 Large vessels and capillaries
(per filed)
 Capillaries (per filed)

## Slide 3
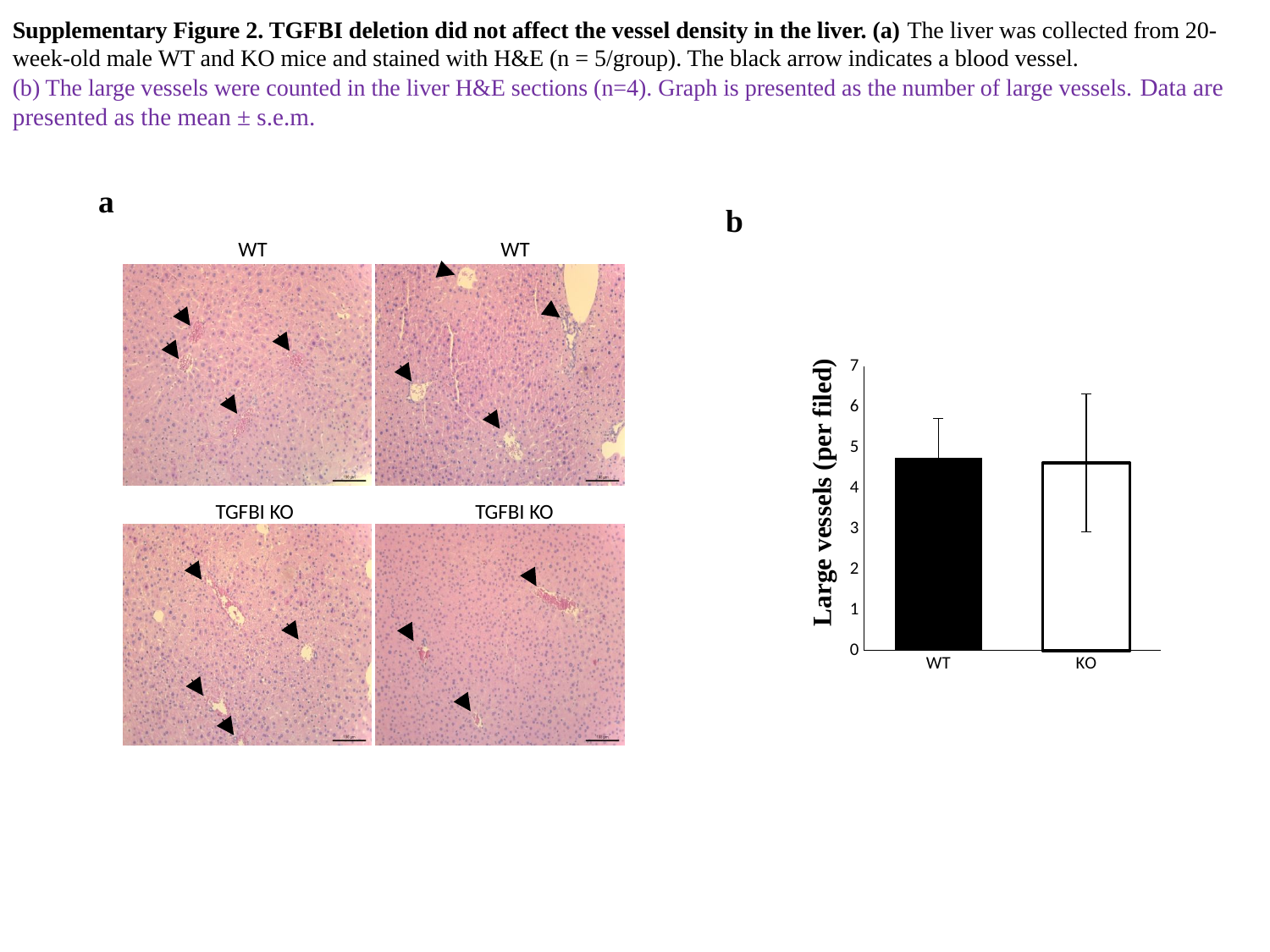

Supplementary Figure 2. TGFBI deletion did not affect the vessel density in the liver. (a) The liver was collected from 20-week-old male WT and KO mice and stained with H&E (n = 5/group). The black arrow indicates a blood vessel.
(b) The large vessels were counted in the liver H&E sections (n=4). Graph is presented as the number of large vessels. Data are presented as the mean ± s.e.m.
a
b
WT
WT
TGFBI KO
TGFBI KO
### Chart
| Category | |
|---|---|
| WT | 4.75 |
| KO | 4.625 |Large vessels (per filed)

## Slide 4
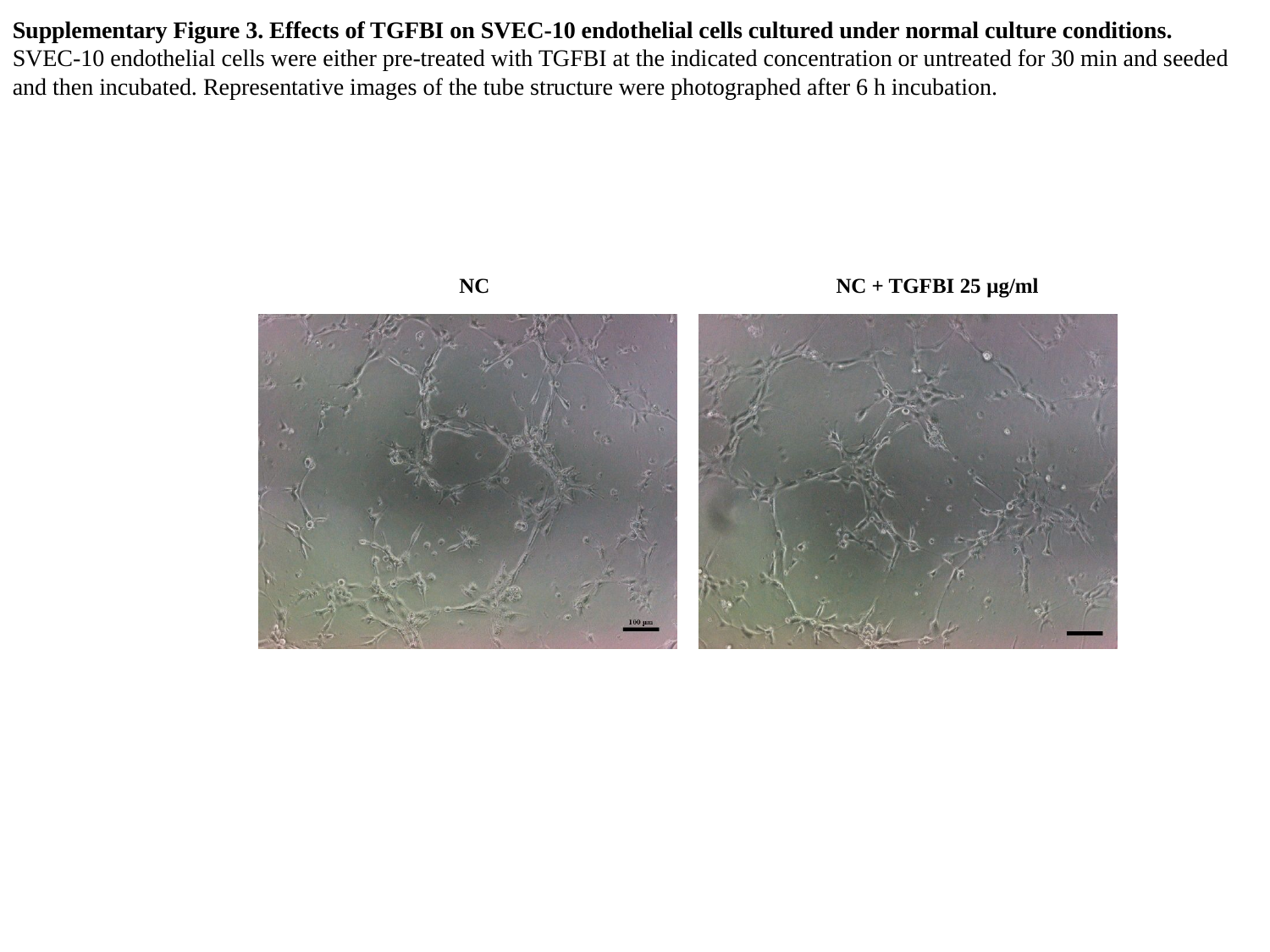

Supplementary Figure 3. Effects of TGFBI on SVEC-10 endothelial cells cultured under normal culture conditions. SVEC-10 endothelial cells were either pre-treated with TGFBI at the indicated concentration or untreated for 30 min and seeded and then incubated. Representative images of the tube structure were photographed after 6 h incubation.
NC
NC + TGFBI 25 μg/ml

## Slide 5
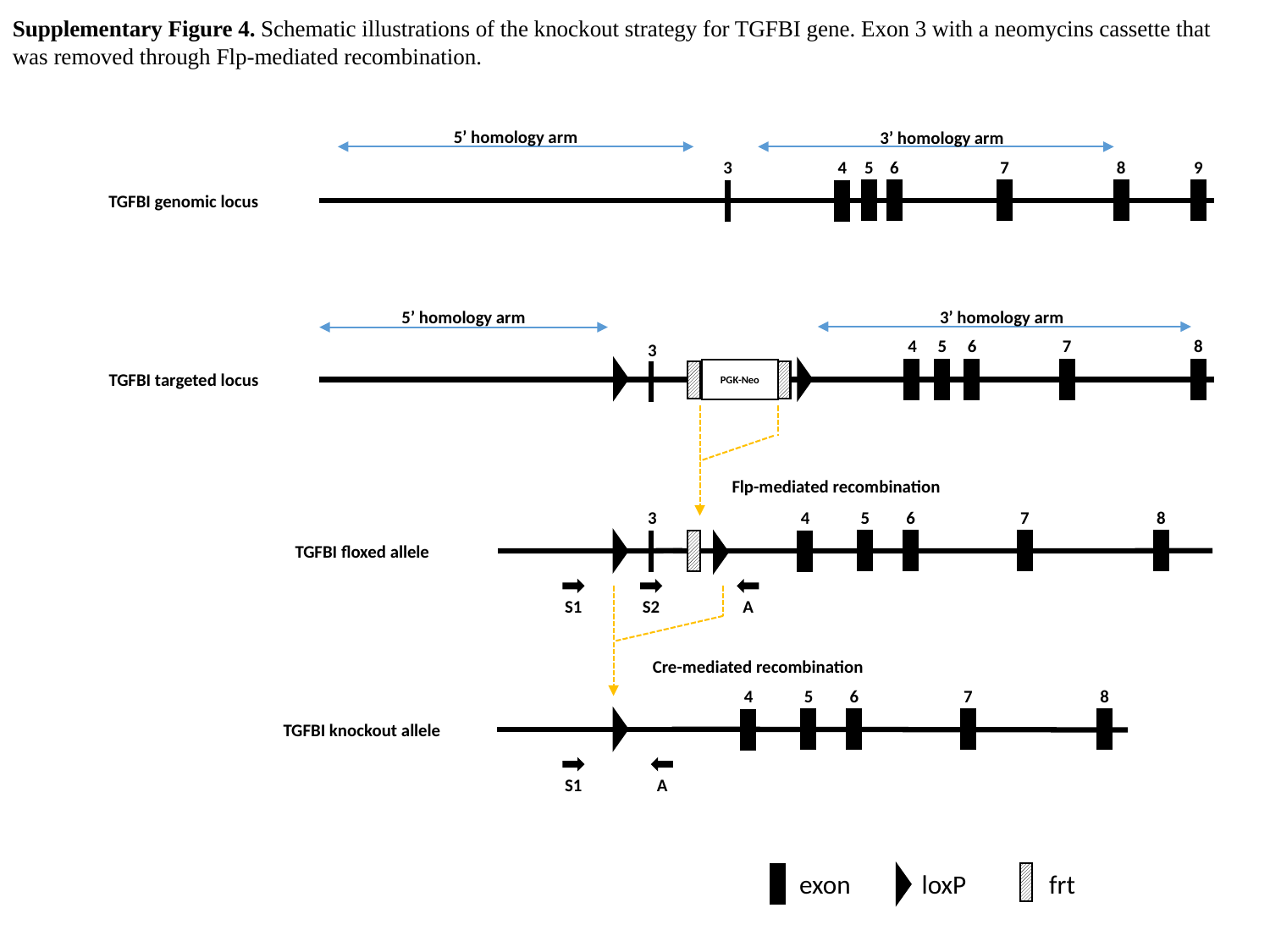

Supplementary Figure 4. Schematic illustrations of the knockout strategy for TGFBI gene. Exon 3 with a neomycins cassette that was removed through Flp-mediated recombination.
5’ homology arm
3’ homology arm
4
5
6
7
8
7
8
TGFBI genomic locus
3
9
3
5’ homology arm
3’ homology arm
4
5
6
PGK-Neo
TGFBI targeted locus
Flp-mediated recombination
4
5
6
7
8
S1
S2
A
Cre-mediated recombination
4
5
6
7
8
S1
A
TGFBI floxed allele
3
TGFBI knockout allele
 exon loxP frt

## Slide 6
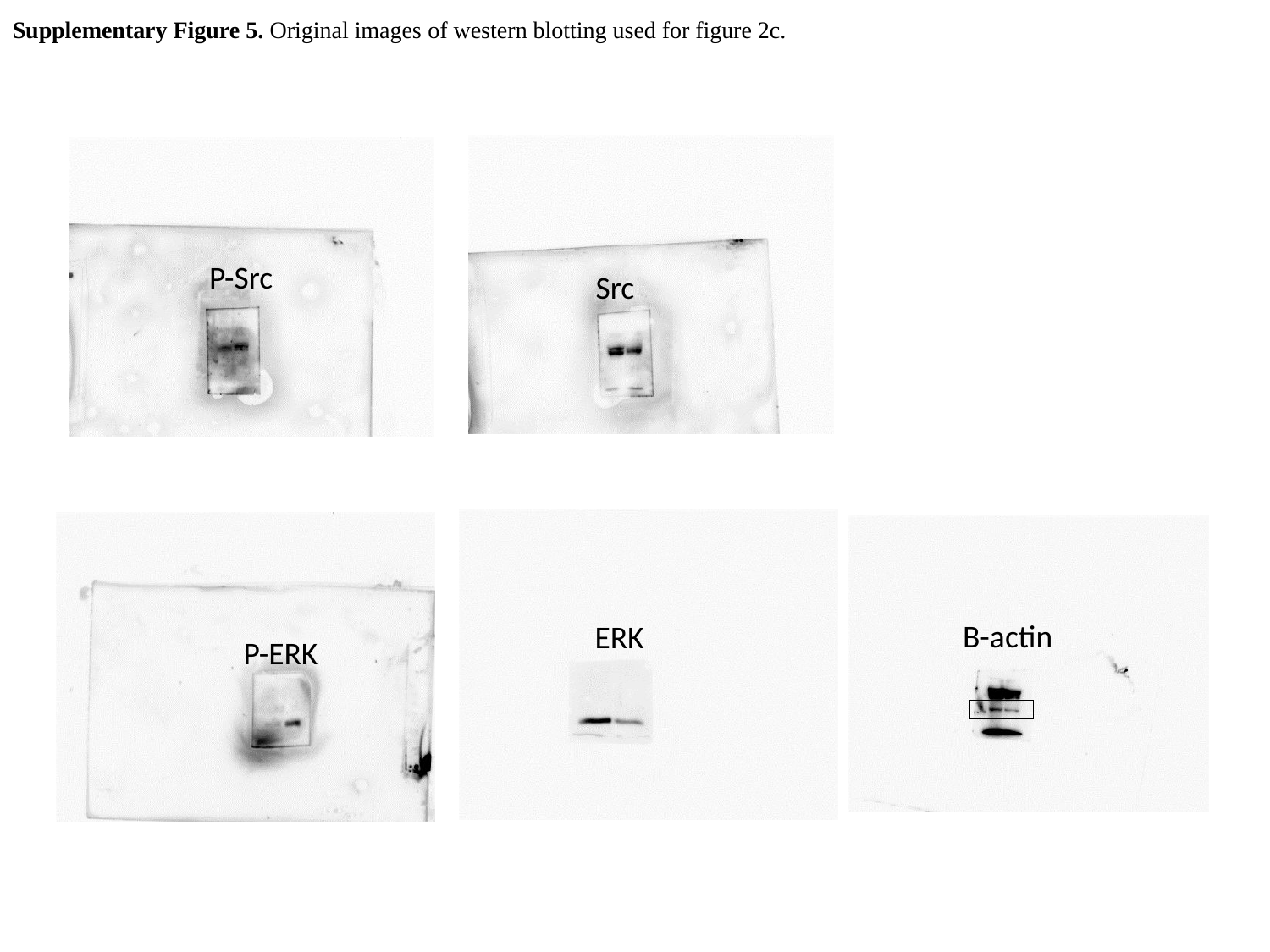

Supplementary Figure 5. Original images of western blotting used for figure 2c.
P-Src
Src
B-actin
ERK
P-ERK

## Slide 7
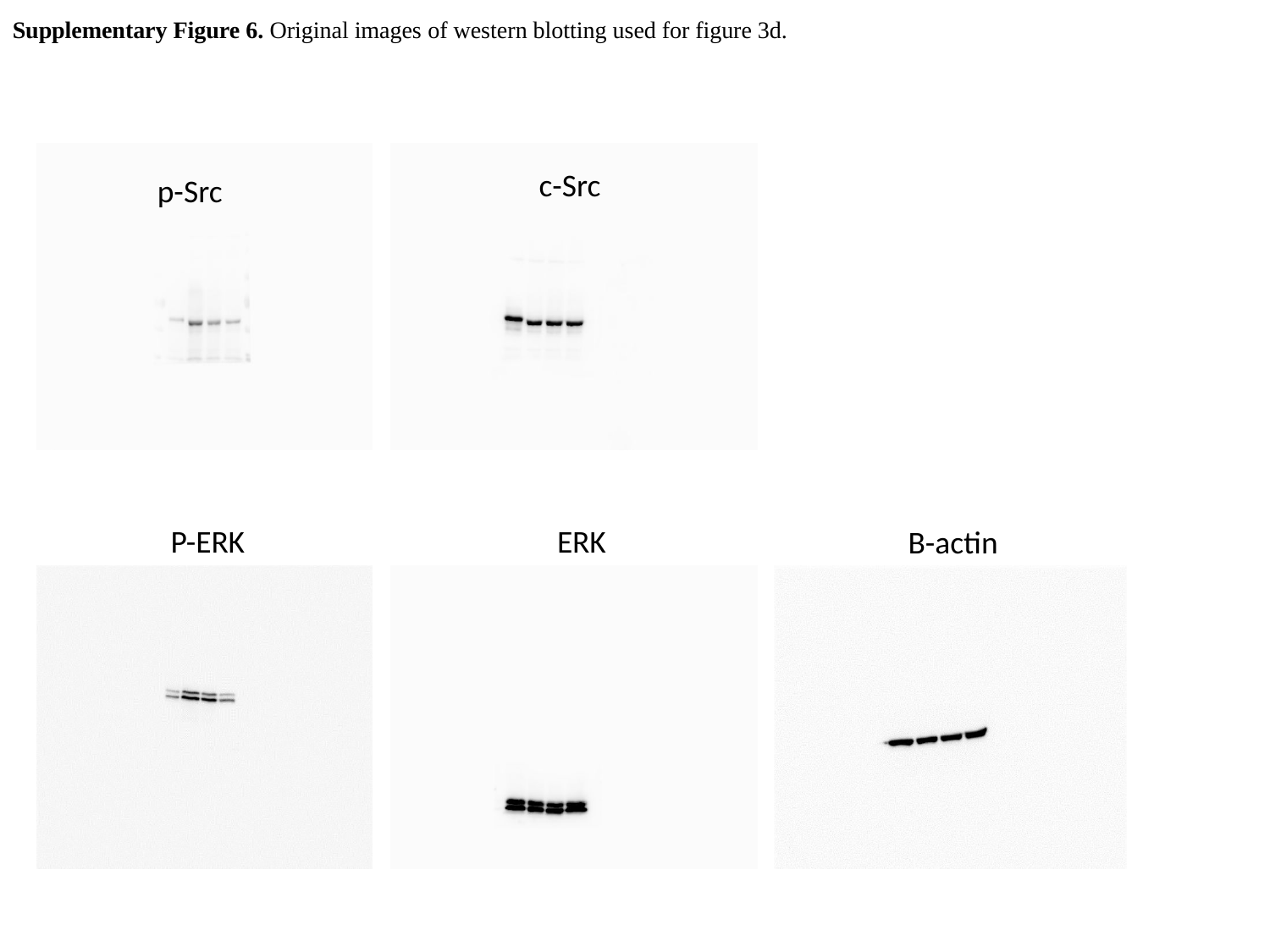

Supplementary Figure 6. Original images of western blotting used for figure 3d.
c-Src
p-Src
P-ERK
ERK
B-actin
